# Trends of *Staphylococcus aureus* bloodstream infections in a neonatal intensive care unit from 2000-2009

**DOI:** 10.1186/1471-2431-14-121

**Published:** 2014-05-09

**Authors:** Olajide Dolapo, Ramasubbareddy Dhanireddy, Ajay J Talati

**Affiliations:** 1 Department of Pediatrics, Division of Neonatology, University of Tennessee Health Science Center, Suite 201, 853 Jefferson Avenue, Memphis, TN 38163-0001, USA

**Keywords:** *Staphylococcus aureus*, Methicillin-sensitive, Methicillin-resistant, Bloodstream, Pneumonia, Sepsis

## Abstract

**Background:**

Invasive methicillin-resistant *Staphylococcus aureus* (MRSA) and methicillin-sensitive *Staphylococcus aureus* (MSSA) infections are major causes of numerous neonatal intensive care unit (NICU) outbreaks. There have been increasing reports of MRSA outbreaks in various neonatal intensive care units (NICUs) over the last decade. Our objective was to review the experience of *Staphylococcus aureus* sepsis in our NICU in the last decade and describe the trends in the incidence of *Staphylococcus aureus* blood stream infections from 2000 to 2009.

**Methods:**

A retrospective perinatal database review of all neonates admitted to our NICU with blood cultures positive for *Staphylococcus aureus* from (Jan 1^st^ 2000 to December 31^st^ 2009) was conducted. Infants were identified from the database and data were collected regarding their clinical characteristics and co-morbidities, including shock with sepsis and mortality. Period A represents patients admitted in 2000-2003. Period B represents patients seen in 2004-2009.

**Results:**

During the study period, 156/11111 infants were identified with *Staphylococcus aureus* blood stream infection: 41/4486 (0.91%) infants in Period A and 115/6625 (1.73%) in Period B (*p* < 0.0004). Mean gestation at birth was 26 weeks for infants in both periods. There were more MRSA infections in Period B (24% vs. 55% *p* < 0.05) and they were associated with more severe outcomes. In comparing the cases of MRSA infections observed in the two periods, infants in period B notably had significantly more pneumonia cases (2.4% vs. 27%, *p* = 0.0005) and a significantly higher mortality rate (0% vs. 15.7%, *p* = 0.0038). The incidences of skin and soft tissue infections and of necrotizing enterocolitis were not significantly changed in the two periods.

**Conclusion:**

There was an increase in the incidence of *Staphylococcus aureus* infection among neonates after 2004. Although MSSA continues to be a problem in the NICU, MRSA infections were more prevalent in the past 6 years in our NICU. Increased severity of staphylococcal infections and associated rising mortality are possibly related to the increasing MRSA infections with a more virulent community-associated strain.

## Background

Treatment of *Staphylococcus aureus* infections in the neonatal intensive care unit (NICU) continues to be a high priority, and reducing the burden of all staphylococcal infections remains of utmost importance. Invasive methicillin-sensitive (MSSA) and methicillin-resistant (MRSA) *Staphylococcus aureus* bloodstream infections in the newborn present with a wide range of serious complications. The situation is particularly worse in the preterm infant, where the developmental immaturity of the immune system increases the susceptibility to these infections. Complications may include brain or visceral abscesses, meningitis, orbital cellulitis, osteomyelitis, septic arthritis, endocarditis, pneumatoceles and lung abscesses, septic ileus, septic shock and, not infrequently, death [1-5]. Numerous recent outbreaks in the NICUs have been attributed to strains of MRSA found both in the health care environment and in the community. The emergence of community-associated methicillin-resistant *Staphylococcus aureus* (CA-MRSA) strains in NICU outbreaks has been widely documented [2,4,6-9]. Earlier studies have stressed the differences between the two different strains of MRSA originating from the hospital environment and from the community, but there are little data available emphasizing the potential change in the epidemiological trend of *Staphylococcus aureus* blood stream infections in the NICU, with increasing reports of MRSA outbreaks [1,4,8,10-13].

There were reports by the Center for Disease Control (CDC) in the United States showing a series of outbreaks in the NICU of potential new strains of community-acquired MRSA in and around the year 2004 [1,6]. This was also corroborated by Healy *et al.*[4] in their study in the same period [4].

This study identifies the unique epidemiological characteristics and trends in the incidence of *Staphyloccus aureus* blood stream infections in neonates, with a view to developing strategies to further decrease the risks of infection. We reviewed our data from 2000-2009, and divided it into cohorts based on references to the increased incidence of MRSA in 2004 in several NICUs.

## Methods

This was a retrospective study carried out in a level III NICU in Memphis, Tennessee, USA – The Regional Medical Center at Memphis. The study was done in a 70-bed NICU with a median annual admission rate of 1110 (range 1006 – 1200) admissions per year during the study period (2000-2009). Very low birth weight (VLBW) infant admission rate was about 200 per year. The study was approved by the hospital Institutional Review Board (Reference: 11-01514-XM). The NICU perinatal database was used to create a list of infants hospitalized in the NICU with positive blood culture for *Staphylococcus aureus* (both MSSA and MRSA) in this period. A chart review of all neonates admitted to the NICU with staphylococcal blood stream infection from January 1^st^ 2000 to December 31^st^ 2009 was done.

Subjects were classified into two groups based on the date of hospital admission using the year 2004 as a reference point, which was the year from which earlier reports of MRSA outbreaks in the NICU were documented.

We compared the demographics, clinical characteristics and outcomes of staphylococcal blood stream infections in the periods before and after reported outbreaks of MRSA in the NICU over the last decade. Period A represents infants admitted from January 1^st^ 2000 to December 31^st^ 2003, and Period B comprises infants admitted from January 1^st^ 2004 to December 31^st^ 2009.

### Study design

Data such as gestational age, birth weight, sex, age at diagnosis with a positive blood culture for *S. aureus*, duration of hospitalization, mechanical ventilation and therapy for respiratory distress syndrome, and use of invasive procedures (including umbilical catheterizations and central venous catheter placements) were collected for the study. Clinical features including pneumonia, skin and soft tissue infections and complications of infection (such as occurrence of septic shock and mortality) were included in the data.

*Staphylococcus aureus* infection or colonization of other body sites, such as skin, anterior nares, conjunctiva, etc., without concomitant positive bloodstream cultures were excluded from the study.

Data regarding antibiotic susceptibility patterns were collected for the following antibiotics – penicillin, oxacillin, vancomycin and clindamycin. Inducible resistance to clindamycin by the D-zone test was performed on isolates with erythromycin resistance and clindamycin susceptibility. Isolates were categorized into susceptible and resistant groups.

### Definitions of variables

The diagnosis of pneumonia was considered if clinical criteria were met (acute clinical deterioration, pulse oximetry, increased respiratory support requirement), radiological findings (presence of new or changing infiltrate on chest radiography) and laboratory parameters (elevated C-reactive protein or abnormal white cell count) suggestive of bacterial infection.

Necrotizing enterocolitis (NEC) was only considered if there were features of stage II NEC or higher, based on modified Bell’s criteria [14].

Skin or soft tissue infections were identified based on the individual clinical team’s evaluation and diagnosis. Septic shock was defined as the occurrence of hypotension with evidence of sepsis in the presence of a positive blood culture, with or without signs of end-organ dysfunction. It was also identified as shock occurring within 48 hours of positive blood culture. Mortality related to sepsis was considered if it occurred within 14 days of positive culture. Infection rates were expressed as the number of infants infected per 1000 NICU admissions.

Statistical analyses were carried out using chi squared tests to compare categorical variables between groups and the extended Mantel-Haenszel chi squared test for linear trend [15] was used to analyze the trend data. Continuous variables were compared using medians of variables and the interquartile range. Statistical significance was set at *p* < 0.05.

## Results

During the study period, 156 (1.4%) of 11,111 NICU infants were identified with *Staphylococcus aureus* blood stream infection. Period A (Jan 1^st^ 2000 – Dec 31^st^ 2003) had 41 (0.91%) cases out of 4,486 total NICU admissions, while Period B (Jan 1^st^ 2004 – Dec 31^st^ 2009), had significantly higher number with 115 (1.73%) cases, of a total of 6,625 infants (*p* = 0.004).

In 2007, education on hygiene and hand-washing methods was intensified and the use of vancomycin locks was introduced (later discontinued in 2009). Otherwise, there were no other changes in the care provided in the two study periods. The total length of stay for VLBW infants in our NICU did not seem to change over time and ranged between 48-61 days mean duration, being 54.7 days in 2000 and 61.6 days in 2009.

As shown in Table 1, the median birth weight and gestation of infants in both periods, irrespective of MSSA or MRSA infection, were similar. The frequency of exposure to invasive procedures and devices was also identical in the two periods (87.8% vs 87.8%) p = 1.000. Mean duration of umbilical catheter days was similar (7.89 ± 6.62 days vs. 7.10 ± 7.23 days) p = 0.543. There was no significant difference in the mechanical ventilation requirements of cohorts in both periods (92.7% vs. 93.0%) p = 1.000. Table 2 shows the sepsis-related mortality in different birth weight groups with both MRSA and MSSA infections. The risk for mortality does not decrease with increasing birth weight with MRSA infections (*p* = 0.16) as compared to MSSA, where mortality was significantly lower with increasing birth weight (*p* < 0.05).

**Table 1:** **Characteristics of infants with *Staphylococcus aureus *infection during the two study periods**

	**Period A (n = 41)**		**Period B (n = 115)**	
**Characteristics**	**MSSA (n = 31)**	**MRSA (n = 10)**	**MSSA (n = 51)**	**MRSA (n = 64)**
Birth weight (g)				
Median (25^th^-75^th^ percentile)	752 (553-977)	737 (563-1120)	838 (647-1081)	736 (580-945)
Category, n (%)				
<750	16 (52)	4 (40)	21 (42)	37 (58)
751 – 1000	7 (23)	0 (0)	14 (28)	17 (26)
1001 – 1250	2 (6)	2 (20)	8 (16)	4 (6)
1251 – 1500	2 (6)	2 (20)	2 (4)	1 (2)
>1500	4 (13)	2 (20)	5 (10)	5 (8)
Gestational age (weeks)				
Median (25^th^-75^th^ percentile)	27 (25-29)	27 (24-31)	27 (26-30)	27 (25-29)
Category, n (%)				
23-25	9 (29)	3 (30)	9 (18)	20 (31)
26-28	12 (39)	3 (30)	25 (48)	23 (36)
29-31	6 (19)	2 (20)	11 (22)	13 (20)
≥32	4 (13)	2 (20)	6 (12)	8 (13)
Gender				
Male (%)	12 (39)	5 (50)	26 (51)	32 (50)
Female (%)	17 (61)	5 (50)	25 (49)	32 (50)
Frequency of invasive procedures				
n (%)	27 (87)	9 (90)	46 (90)	55 (86)
Mechanical ventilation				
n (%)	29 (94)	9 (90)	47 (92)	60 (94)
Age at diagnosis (days)				
Median	27	25	22	25

**Table 2:** Survival rates among infants during the two study periods

**Birth weight distribution (grams)**	**Period A (N = 41)**		**Period B (N = 115)**	
	**MSSA (n = 31)**	**MRSA (n = 10)**	**MSSA (n = 51)**	MRSA (n = 64)*
<750	16	4	21	37
Survived (%)	12 (75)	4 (100)	17 (81)	26 (70)
750 – 999	7	0	14	17
Survived (%)	7 (100)	0	12 (86)	12 (71)
1000 – 1499	2	2	8	4
Survived (%)	2 (100)	2 (100)	8 (100)	3 (75)
1500 - 1999	2	2	2	1
Survived (%)	2 (100)	2 (100)	2 (100)	0 (0)
≥ 2000	4	2	5	5
Survived (%)	4 (100)	2 (100)	5 (100)	(100)

*Trend analysis of survival rates for MRSA and MSSA infections in the weight categories, using the extended Mantel-Haenszel chi-square for linear trend, showed a significant risk of death when weight was <750 grams for MSSA cases (*p* = 0.0166), but no significant survival trend with increasing gestational age seen in MRSA cases.

MRSA infections were significantly higher in Period B (24% vs. 55%, *p* < 0.05) and, as shown in Table 3, were also associated with more severe outcomes. In comparing the cases of MRSA infections observed in these two periods, infants in period B notably had a significantly higher incidence of pneumonia (2.4% vs. 27%, *p* = 0.0005) and a significantly higher mortality rate (0% vs. 15.7%, *p* = 0.0038). The incidences of skin and soft tissue infections and that of necrotizing enterocolitis were not significantly different in the two periods. Period B was associated with an increasing trend of septic shock complications, although this was not statistically different from Period A.

**Table 3:** **Complications of *Staphylococcus aureus *blood stream infections**

	**Period A (2000-2003) n = 41**		**Period B (2004-2009) n = 115**		***p *value *(comparing MRSA infection in the two periods)***
***Complications***	**MSSA (n = 31)**	**MRSA (n = 10)**	**MSSA (n = 51)**	**MRSA (n = 64)**	
Septic shock	0 (0%)	1 (2.4%)	4 (3.5%)	13 (11.3%)	0.115
Concomitant soft tissue/skin infection	4 (9.8%)	6 (14.6%)	5 (4.3%)	13 (11.3%)	0.584
Pneumonia	6 (14.6%)	1 (2.4%)	7 (6.1%)	31 (27.0%)	0.0005
Necrotizing enterocolitis	1 (2.4%)	4 (9.8%)	3 (2.6%)	5 (4.3%)	0.2435
Mortality	4 (9.8%)	0 (0%)	6 (5.2%)	18 (15.7%)	0.0038
Length of hospitalization (mean number of days)	87.4 ± 40.6	80.6 ± 42.4	55.7 ± 30.2	53.6 ± 36.6	0.0371

MRSA-infected infants in period B had a significantly shorter mean length of hospitalization than similarly infected infants in period A (80.6 ± 42.39 vs. 53.6 ± 36.6 total hospital days; *p* = 0.0371). Infants with MSSA were also noted to have a much shorter hospital course in Period B (87.4 ± 40.6 vs. 55.7 ± 30.2 days; *p* = 0.0001).

**Figure 1:**
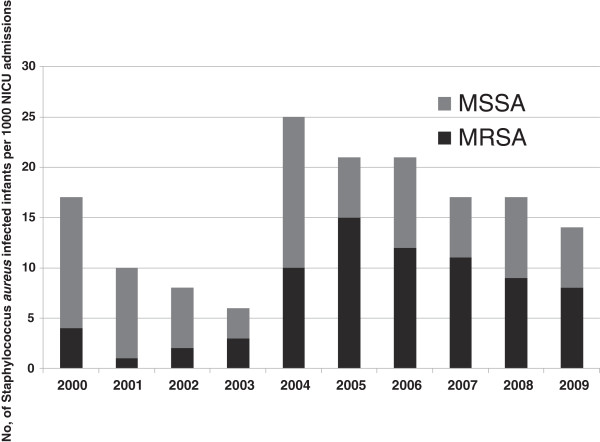
**The yearly trend of MSSA and MRSA infection in the last decade, showing a significant rise in overall incidence of *Staphylococcus aureus *infections in 2004.** The extended Mantel-Haenszel chi-square test for linear trend also showed a significant increase in MRSA infections over the 10-year period (*p* = 0.0007), but no trend in increase of MSSA infections (*p* = 0.229).

The yearly trend of MRSA versus MSSA infections, with the number of infected infants per 1000 NICU admissions, is shown in the Figure 1. This shows an overall rise in the incidence of *Staphylococcus aureus* blood stream infections from the year 2004 in our NICU. Analyses of the trend data for MSSA and MRSA infections over the study period were performed using the extended Mantel-Haenszel chi-squared test for linear trend. Results demonstrated a significant increase in trend for MRSA infections, but not for MSSA infections. (MRSA trend analysis *p* = 0.000702 vs. MSSA *p* = 0.229).

All *Staphylococcus aureus* isolates (MSSA and MRSA) were susceptible to vancomycin. The sensitivity pattern of MRSA to clindamycin was similar in the two periods: 60% of MRSA isolates were sensitive to clindamycin in Period A vs. 64% in Period B.

## Discussion

According to a 2011 CDC report, the incidence of MRSA in the community in general has increased rapidly in the past decade, with little or no evidence of recent decline, despite clear evidence that invasive MRSA infections in the health care setting is declining [6]. The implementation of aggressive infection control techniques in the health care environment has proved successful in reducing the incidence of health care-associated infections in various NICUs [8]. Our study demonstrates a rise in the overall incidence of *Staphylococcus aureus* blood stream infections observed in the NICU in the last 10 years, with a peak period around the year 2004. This period coincides with widespread reports of CA-MRSA outbreaks in the NICUs [1,2,4,5,8]. The incidence of MRSA infections in the NICU is still unacceptably high, and this may be likely linked to the acquisition of CA-MRSA strains, which have evolved in the community and penetrated the NICU through either parents or care providers of the patient [8,9,16-19].

During the study period we detected that significantly more MRSA infections were seen in the last 6 years, and that these cases were more frequently associated with severe clinical presentations and worse outcomes. In previous studies, there was earlier onset of MRSA infections compared to MSSA infections, which was attributed to possible vertical transmission of infection [16]. This was not, however, the finding in our study, where the median age at presentation for MSSA infections was 27 days in Period A and 22 days in Period B, while the median age at diagnosis for MRSA infections was 25 days in the two periods.

The rate of skin and soft tissue infections was not significantly different for either MRSA or MSSA cases during the two periods reviewed in the study. Similar findings were reported by Carey *et al.*, who compared 123 infections caused by MSSA and 49 caused by MRSA in a neonatal ICU. The rate of skin and soft tissue infections was similar for both groups at 45% [16]. However, in our study, there may have been an underestimation of skin and soft tissue infection cases, as those without positive blood culture were excluded from the study.

Duration of hospital stay in the second period of our study was significantly less than in the initial 4-year period. It is unclear whether the increased incidence of MRSA infections with more severe complications in the subsequent 6 years led to increased mortality or whether an improvement in neonatal care and management approach led to shorter hospital stay for ELBW infants. An earlier study by Burke *et al.* found 164 episodes of *S. aureus* bacteremia in 151 children and infants [3]. In this study, children with MRSA infection stayed in the hospital longer (with a mean of 36 days) than did children with MSSA infection (mean 16.3 days). However, the study was done in a cluster of not just neonates, but children and infants.

In our study, the predominant weight category of all infants with *Staphylococcus aureus* blood stream infection in the NICU was noted to be less than 750 g (51% of all cases), and they were extremely preterm. Reasons for this were described by Healy *et al.*[4] in an earlier study, emphasizing risk factors for staphylococcal infections that are peculiar to extremely low birth weight infants; namely, poorly developed host defense mechanisms, central venous catheter requirements, need for endotracheal or upper gastrointestinal tube placement, and procedures that might compromise skin integrity. However, a trend analysis of mortality revealed no change in risk with increasing birth weight in MRSA infections. The risk of death was significantly higher in infants < 750 g birth weight, with MSSA infections. Shane *et al.*[20] study, demonstrated no significant difference in morbidity or mortality of very low birth weight (VLBW) infants with MRSA compared with those with MSSA bacteremia. This conclusion probably reflected the multi-center nature of their study, as 40% (8 out of 20) of the study centers, actually reported zero cases of MRSA infection. This also probably demonstrated the variability in the population and practice of these study centers.

The exposure of all infected infants in our study, to risk factors was assessed (such as device utilization and exposure to invasive procedures) and no difference was found between study periods in the degree of exposure to risk factors.

Emphasis remains on infection control practices and the prevention of transmission in identified cases. The importance of judicious compliance to standard infection control practices such as hand hygiene, gloving, protection of eyes, nose and mouth; gowning and appropriate handling of patient care equipment and devices cannot be over-emphasized. Contact precautions must be adhered to in all identified cases [6,7].

Our study emphasizes the changing pattern of *S. aureus* infection in our NICU in the last decade as it relates to increasing reports of MRSA outbreaks. This study is, however, limited by the inability to determine the pathological characteristics and phage –type of isolates, as data were retrospectively collected. The retrospective nature of data collection inherently led to some diagnostic biases. Other limitations that are commonly associated with retrospective chart reviews, such as incomplete documentation, missing data and problematic verification of information are also possible with this study.

## Conclusions

In conclusion, there was an increase in the incidence of *S.aureus* blood stream infections among neonates after 2003, which coincides with increasing reports of MRSA infections in the NICU. Though, MSSA continues to be a problem in the NICU, MRSA infections were more prevalent in the last 6 years. The increased severity of *S aureus* infection and associated rising mortality rate may be related to increasing MRSA infections with a more virulent community-associated strain.

## Abbreviations

NICU: Neonatal intensive care unit; MSSA: Methicillin-sensitive *Staphylococcus aureus*; MRSA: Methicillin-resistant *Staphylococcus aureus*; CDC: Centers for disease control and prevention; ELBW: Extremely low birth weight; VLBW: Very low birth weight.

## Competing interest

The authors declare that they have no competing interest.

## Authors’ contributions

OD developed the study concept, designed the study and drafted the manuscript. RD participated in the design of the study and performed the statistical analysis. AT conceived the study, participated in its design, coordination, and statistical analysis and also helped to draft the manuscript. All authors read and approved the final manuscript.

## Pre-publication history

The pre-publication history for this paper can be accessed here:

http://www.biomedcentral.com/1471-2431/14/121/prepub
